# Evaluation of an experiential training program in patient-centered outcomes and comparative effectiveness research for diverse researcher communities and health care organizations

**DOI:** 10.1017/cts.2023.36

**Published:** 2023-03-15

**Authors:** Dedra S. Buchwald, Megan H. Rogers, Barbara A. Rose, Brian W. Bresnahan, Beth Devine, Linda LeResche, Lonnie A. Nelson, Donald L. Patrick, Danielle C. Lavallee, Michelle M. Garrison, Andrew A. White, Larry G. Kessler

**Affiliations:** 1 From the Washington State University Institute for Research and Education to Advance Community Health, Seattle, WA, USA; 2 Elson S. Floyd College of Medicine, Pullman, WA, USA; 3 Department of Health Systems and Population Health, University of Washington School of Public Health, Seattle, WA, USA; 4 Department of Pharmacy, School of Pharmacy, Seattle, WA, USA; 5 Department of Radiology, School of Medicine, Seattle, WA, USA; 6 Department of Oral Medicine, School of Dentistry, Seattle, WA, USA; 7 Washington State University College of Nursing, Seattle, WA, USA; 8 Departments of Surgery, University of Washington School of Medicine, Seattle, WA, USA; 9 Departments of Psychiatry, University of Washington School of Medicine, Seattle, WA, USA; 10 Departments of Medicine, University of Washington School of Medicine, Seattle, WA, USA

**Keywords:** Comparative effectiveness, patient-centered outcomes, training programs, mentoring, American Indian and Alaska Native

## Abstract

**Background/Objective:**

The goal of the Patient-Centered Outcomes Research Partnership was to prepare health care professionals and researchers to conduct patient-centered outcomes and comparative effectiveness research (CER). Substantial evidence gaps, heterogeneous health care systems, and decision-making challenges in the USA underscore the need for evidence-based strategies.

**Methods:**

We engaged five community-based health care organizations that serve diverse and underrepresented patient populations from Hawai’i to Minnesota. Each partner nominated two in-house scholars to participate in the 2-year program. The program focused on seven competencies pertinent to patient-centered outcomes and CER. It combined in-person and experiential learning with asynchronous, online education, and created adaptive, pragmatic learning opportunities and a Summer Institute. Metrics included the Clinical Research Appraisal Inventory (CRAI), a tool designed to assess research self-efficacy and clinical research skills across 10 domains.

**Results:**

We trained 31 scholars in 3 cohorts. Mean scores in nine domains of the CRAI improved; greater improvement was observed from the beginning to the midpoint than from the midpoint to conclusion of the program. Across all three cohorts, mean scores on 52 items (100%) increased (p ≤ 0.01), and 91% of scholars reported the program improved their skills moderately/significantly. Satisfaction with the program was high (91%).

**Conclusions:**

Investigators that conduct patient-centered outcomes and CER must know how to collaborate with regional health care systems to identify priorities; pose questions; design, conduct, and disseminate observational and experimental research; and transform knowledge into practical clinical applications. Training programs such as ours can facilitate such collaborations.

## Introduction

Health care systems face complex issues that raise difficult questions and create challenges in providing high-quality care in a resource-constrained environment. Are we generating and effectively using evidence on the relative benefits and harms of clinical and public health interventions? How certain are we that our health care and behavioral decisions improve a population’s health? How quickly can the research community adapt to new challenges, such as those posed by the COVID-19 pandemic? Answering these questions requires high-quality, multidisciplinary research focused on outcomes that matter to patients and other stakeholders, such as providers and policymakers. To achieve these ends, the USA must nurture and invest in a cadre of new investigators as well as those already in the health care workforce who can design and conduct innovative, pragmatic, patient-centered outcomes research (PCOR) and comparative effectiveness research (CER). Further, training diverse investigators and integrating health equity into learning health systems research is imperative [1]. Such investigators and their health care organizations must be poised to implement these two closely related types of research in varied and often underfunded health care settings.

According to the Patient-Centered Outcomes Research Institute (PCORI), PCOR focuses attention on patients’ beliefs, preferences, and needs, to address research questions that matter to patients; it emphasizes active patient participation at all stages as an essential element of research [2,3]. The Agency for Healthcare Research and Quality (AHRQ) defines CER as research that compares the results of one approach for managing a disease to the results of other approaches, for example, use of different drugs for the same disease [4]. One purpose of CER is to provide information that helps clinicians and patients choose the option that best fits an individual patient’s needs and preferences [5]. More broadly, CER includes conducting original research or systematic reviews of published literature to compare the benefits and risks of different approaches to preventing, diagnosing, or treating diseases; this includes studies of health care systems and/or interventions implemented within them.

The American Recovery and Reinvestment Act of 2009 allocated $1.1 billion for CER to federal agencies including AHRQ [3]. In 2013, AHRQ released a funding opportunity announcement for an R25 training program on “Researcher Training and Workforce Development in Methods and Standards for Conducting Patient-Centered Outcomes Research Studies” [6]. The announcement required the applicant institution to work with partners to build capacity through a multicomponent education program with basic, advanced, and experiential training. AHRQ selected five institutions with innovative platforms (detailed elsewhere [7]), including the University of Washington (UW), to train clinician-scientists in PCOR and CER.

Since 2013, PCORI and AHRQ have been building research capacity in PCOR and learning health systems as reflected by their portfolio of funded K12 training programs [8], which predated the re-authorization of PCORI [9]. In addition, PCORI’s 2022 Strategic Plan emphasizes a commitment to diversity, equity, and inclusion and reiterates PCORI’s “commitment to patient-centeredness, meaningful stakeholder engagement, and supporting ongoing development of the partnerships across the healthcare community” [10]. The goal of the Patient-Centered Outcomes Research Partnership (PCORP) at UW and Washington State University (WSU) was to prepare scientists, clinicians, other health care professionals, and researchers to conduct PCOR and CER in a wide range of settings with regional partners that serve diverse and underrepresented patient populations. We achieved this goal by building an innovative training program that combined in-person and experiential learning with asynchronous, online education driven by the expressed needs and interests of our partners. These partners comprised five community-based health care organizations scattered across an enormous geographic expanse including the five-state WWAMI region (Washington, Wyoming, Alaska, Montana, and Idaho), Minnesota, Hawai’i, and North and South Dakota. The PCORP definition of community may be a bit different than one might see in other program, necessitated by the geography of our program.

We adopted an explicit strategy to augment skills of individuals *already in the health care workforce in community settings*, rather than training a new cadre of clinician and scientist scholars entering the workforce at a single institution. We reasoned that scholars working in their home institutions and sharing lessons learned in real time had advantages compared to traditional programs that require scholars to move to academic institutions to complete training programs. Our approach ensured our community-based clinician and scientist scholars were intimately familiar with the culture, problems, and priorities of their home institutions. Our strategy also helped align the research goals and priorities of the home institution with advancing the skills of both academic and community-based researchers. In addition, PCORP projects were required to directly link to the scholars’ professional responsibilities, which enhanced both the generalizability and the efficiency of implementing their projects’ deliverables in their home institution. Lastly, our scholars were well positioned within their home institutions to identify mentors willing and able to provide the resources and time needed for adequate progress of the PCORP projects.

With the notable exception of the University of Pittsburgh ENACT program[7], few PCOR and CER training programs at the time we launched PCORP included an explicit focus on underrepresented communities and training in real-world conditions. Fortunately, linkages between community-based participatory research and learning health systems now are part of the PCORP and CER landscape [11]. We, therefore, describe the structure, evaluation, and outcomes of the PCORP program and offer comments on lessons learned in its planning and execution to inform future efforts.

## Methods

### Academic Program Structure

PCORP was a collaboration between UW and WSU. Core program faculty were drawn from the Department of Health Services (since changed to Health Systems and Population Health) in the UW School of Public Health, the Comparative Health Outcomes, Policy, and Economics (CHOICE) Institute in the UW School of Pharmacy, the Department of Oral Medicine in the UW School of Dentistry, the Departments of Surgery and Radiology in the UW School of Medicine, and the Institute for Research and Education to Advance Community Health at WSU. The latter conducts community-based research with American Indian, Alaska Native, Native Hawaiian, and Pacific Islanders communities across the nation and offers a variety of career development programs to increase the diversity of the scientific workforce.

Other resources available to PCORP scholars included those of the UW Institute of Translational Health Sciences [12], one of 61 NIH-funded Clinical and Translational Science Awards. The Institute strives to foster collaboration among academic researchers and communities across the WWAMI region. In addition, the nationwide UW Centers for Comparative and Health Systems Effectiveness (CHASE) Alliance brings together successful research groups and community partners to undertake high-impact PCOR and CER and share resources such the CER curriculum used as a foundation for the PCORP program [13].

### Community Organization Partners

PCORP was a stakeholder-driven educational and experiential training program designed to train scientists, clinicians, and other health care professionals involved in quality improvement, research, and evaluation initiatives. Two critical and unique features of PCORP were an emphasis on the specific needs of the partner organizations and its focus on the underserved populations of American Indian, Alaska Native, Native Hawaiian, and Pacific Islander peoples. PCORP scholars came from five community organizations located over vast geographic areas with large rural and minority populations. The *Southcentral Foundation* in Anchorage, Alaska, the medical home for 66,000 Alaska Native and American Indian people [https://www.southcentralfoundation.com/], offers a wide range of health and wellness services for Alaska Native and American Indian people living in Anchorage and the Matanuska-Susitna Borough and nearby villages. Southcentral Foundation also serves residents of 55 rural villages in the Anchorage Service Unit, a geographical area stretching 107,400 square miles across Southcentral Alaska from the Canadian border on the east to the Aleutian Chain and Pribilof Islands on the west. Community members are referred to as customer owners, demonstrating the organization’s commitment to inclusivity in all aspects of care. *Swedish Health Services* (formerly Swedish Medical Center) is the largest nonprofit health care provider in the Seattle metropolitan area. It operates five hospital campuses and ambulatory care centers and includes the Swedish Medical Group, a network of more than 100 primary care and specialty clinics. Through research and education, the *Department of Native Hawaiian Health at the University of Hawai’i at Mānoa* in Honolulu comprises a comprehensive program that addresses the health and health care needs of Native Hawaiian and Pacific Islander people. Based in Sioux Falls, South Dakota, *Sanford Health*, the largest rural health system in the USA, is dedicated to the integrated delivery of world-class health care to more than one million patients across 250,000 square miles. Sanford Health comprises 47 hospitals, 2,800 physicians, and more than 200 senior care locations in 26 states and 10 countries [https://www.sanfordhealth.org/]. The *MultiCare Health System* is a not-for-profit US health care organization based in Tacoma, Washington. Founded in 1882, MultiCare provides health care services at 504 locations, including 11 hospitals, across Washington state. Through the UW’s Institute of Translational Health Sciences, an NIH-funded Clinical and Translational Science Award, community-based faculty from partner organizations and programs in the WWAMI region could participate in PCORP. These included the WWAMI region Practice and Research Network and the Northwest Participant and Clinical Interactions Network, which connect diverse populations to local, high-quality clinical research.

### Application Process and Selection of Participants

PCORP planned to enroll two scholars from each partner organization in each of 3 cohorts, totaling 24 scholars. Candidates primarily had masters or doctoral-level training (e.g., MA, MPH, MD, DNP, PhD) and included both clinicians and nonclinicians. Six months before the program began, applicants submitted a research idea of interest to their home organization that could be addressed by PCOR and CER and identified potential local mentors. Potential PCORP scholars were required to choose feasible topics that were expected to be unsolved for the next 2 years (i.e., the duration of PCORP training) and have 10% time covered by their partner organizations to complete the PCORP program. They also submitted a one-page statement of purpose, a two-page research plan, a CV, and two letters of support, including one from a leader in their institution supporting the applicant and confirming the research idea was a priority for the health system. The application process considered the qualifications of the candidates, their role in the partner organization, and the reasons for their interest in PCOR and CER. The organizational letters of support were evaluated by the PCORP Advisory Board and in at least two instances they were the reason an application was not seemed acceptable.

The PCORP Advisory Board, composed of representatives from each of our partner organizations and three UW faculty with expertise in PCOR and CER, reviewed candidate applications. Inclusion of partner organizations on the evaluation panel ensured engagement and selection of projects meaningful to the organization. The Board considered: 1) readiness to conduct research; 2) understanding of the tools needed to conduct interdisciplinary PCOR and CER; 3) strength of applicant’s research idea; and 4) importance of the research question to the community-based organization to ensure support for the applicant’s project and experiential PCORP training. Successful candidates were selected based on the strength of their applications while striving for diversity in gender, race and ethnicity, and academic discipline.

### Training Program Competencies and Components

The PCORP program was designed to help scholars acquire seven fundamental competencies: 1) describe health services research, evidence-based medicine, and the historical and regulatory evolution of PCOR and CER; 2) articulate methods, findings, and implications of PCOR and CER; 3) critically appraise PCOR and CER studies; 4) identify relevant data sources and their strengths and weaknesses; 5) apply results of PCOR and CER to clinical domains; 6) describe quality metrics and their use in health care and payment models; and 7) describe diverse stakeholders and best practices for engaging patients and stakeholders in all phases of PCOR and CER. Because program partners had different goals and projects, proactively engaging patients in PCORP was difficult. We, therefore, offered an in-person PCORP session that convened a panel on the importance of patient engagement in PCOR projects and encouraged scholars to incorporate patient input into their individual projects. The latter is exemplified by the work of one of our scholars on a national registry [14].

Core program components comprised a tailored PCOR and CER curriculum, offered during an intensive, week-long, in-person institute, augmented by self-paced online-training modules (Table 1). PCORP’s overarching goal was to help scholars develop their pilot projects as the means to engage in experiential training. We also aimed to design an effective mentoring structure that included mentors from the scholars’ home institutions, UW, and WSU, and to create a peer-to-peer training network. The 2-year program began with four modules of online training, which consisted of readings about health services research, both quantitative and qualitative evidentiary methods, health economics, and basic information on PCOR and CER. Each module had a moderated discussion board that connected scholars, their mentors, and other designated core faculty to ensure that scholars could master selected critical concepts before attending the PCORP Summer Institute. These online training components foreshadowed the hybrid learning style that has become common since the beginning of the pandemic [15]. In addition, most of these components are now part of the movement to create competencies in learning health care systems [16–19].

**Table 1.** Example of the PCORP cohort schedule

**April 2016**	**Introduction activities**
	Learning contract, Introductory webinar Complete initial Research Skills Inventory (online survey) Begin work on Individual Development Plan Individual mentor meetings
**April–May 2016**	**Online training**
	Online training: Health Services Research Online training: Overview of PCOR and CER Concepts Online training: Further Topics in CER Online training: Outcomes and Outcomes Assessment
**June 2016**	**Pre-Summer Institute Project Preparation**
	Online webinar to introduce projects Scholars’ project proposals due Abstracts due
**July 11–14, 2016**	**Summer Institute**
**July–August 2016**	**Individual Mentor Meetings**
**Fall 2016–June 2018**	**Ongoing Project Tasks**
	Project work continues (until April 2018) Individual mentor meetings (quarterly) Monthly progress reports commence (beginning Winter 2016) Periodic Online Modules
**June–July 2017, June 2018**	**Program Evaluation Activities**
	Evaluation tracking forms due (see above) Research Skills Appraisal Inventory (online survey) Mentor Interview, Mentee Interview, Mentoring and Advising Report, Faculty Evaluation of Mentee Collaboration Tracking Tool (12 months) (2017) Collaboration Tracking Tool (24 months) (2018)

PCORP, Patient-Centered Outcomes Research Partnership; PCOR, patient-centered outcomes research; CER, comparative effectiveness research.

Next, scholars participated in a week-long PCORP Summer Institute that provided didactic sessions on study design and analytic methods, evidence synthesis, stakeholder engagement, economic analysis, and patient-centered and patient-reported outcomes. It also offered small-group sessions in which panels of experts working in relevant fields discussed topics such as quality improvement data for research, and panels of patient, clinician, administrative, and senior stakeholders elucidated issues they face. Group exercises were used throughout the Institute to provide hands-on experience in PCOR and CER methods relevant to scholars’ projects. In addition, scholars’ projects were extensively and iteratively reviewed by their peers and PCORP faculty during the Summer Institute. Networking during the PCORP Summer Institute was encouraged as the peer review sessions helped strengthen each pilot project’s design and served as an intentional educational tool. Daily feedback sessions and iterative revisions culminated in a final presentation by each scholar describing the revised project proposed as the foundation for their subsequent training.

After the Summer Institute, scholars continued to meet with their home institution, UW, and WSU mentors at least quarterly to ensure adequate progress toward completing the program by the end of their second year. During the second year of scholars’ experiential training, we also developed two modules covering research approaches to working with underrepresented populations and statistical methods and approaches used in PCOR and CER. Scholar assignments after the Summer Institute included additional online modules tailored to their needs, such as PCOR and CER among disadvantaged populations. Examples of scholars’ pilot projects included suicide prevention among Alaska Native people at Southcentral Foundation; health guides to reduce high health care utilization at Sanford Health; and psychosocial aspects of adolescent and young adult cancer survivorship at MultiCare Health System (Sanford and MultiCare connected through WWAMI; Table 2).

**Table 2.** Examples of PCORP pilot projects

Project description	Organization or institution	PCORP scholar role
Health Guide Intervention for High-Risk Patients	Sanford Health (SD)	Assistant Scientist – Co-Investigator on the Environmental Influences on Child Health Outcomes (ECHO) grant
Impact of Primary Care Integrated Behavioral Health and Emergency Department Utilization	Swedish Medical Group (WA)	Program Administrator for Primary Care Integrated Behavioral Health and Swedish Medical Group
Enhancing Family Cohesion through Relationship Awareness among Committed Alaska Native and American Indian Couples	Southcentral Foundation (AK)	Researcher: Piloting a web-based intervention designed to help manage trauma-related behavioral health symptoms
Health Care Homes for Native Hawaiians	University of Hawai’i at Mānoa (HI)	Assistant Professor/Director of Behavioral Health
Understanding Time to Diagnosis and Time to Treatment in Adolescents and Young Adults with Cancer: Scenario in a Community Cancer Care Context	MultiCare Institute for Research and Innovation (WA)	Senior Research Epidemiologist
Pain management and long-acting opioids in reducing pain among American Indian and Alaska Native people	Southcentral Foundation (AK)	Senior Researcher and Medical Services Division Research Team lead
Radiology decision support, pediatric ultrasounds for appendicitis	Swedish Medical Group (WA)	Medical Director of OBX Seattle Ultrasound Associates (Swedish)
Establishing a Child-Centered Research Community that Fosters Long-term Healthy Lifestyles of Children with Disabilities	University of Washington (WA)	Physical Therapist and Acting Assistant Professor in the Department of Rehabilitation Medicine
Chronic Pain Interprofessional Medical Group Visits in Primary Care	Providence Family Medicine Center (AK)	Director of Behavioral Health
Exploring Palliative Care Communication with Alaska Native/American Indian People at Two Primary Care Sites	Southcentral Foundation (AK)	Senior Researcher
Health Care Utilization Outcomes of Healing Childhood Trauma	Southcentral Foundation (AK)	Program Evaluator
Impact of Free Drug Patient Assistance Program on High Cost IV Drugs in Cancer Treatments	Kaiser Permanente (WA)	Clinical Research Specialist/Nurse
Scoping Review of Unmet Family Planning Need among Refugees and Immigrants in the USA	University of Washington (WA)	Assistant Professor in the Department of Family Medicine

PCORP, Patient-Centered Outcomes Research Partnership.

Because AHRQ’s funding announcement required offering advanced training for PCORP scholars with considerable biostatistical background, we developed a training program on machine learning for observational data, in collaboration with the UW Department of Biostatistics and a faculty member at Emory University. Three PCORP Summer Institute scholars attended this program, which has been successfully sustained as part of the UW Summer Institute in Statistics for Clinical and Epidemiological Research.

### Evaluation Measures

We used both qualitative and quantitative measures to evaluate PCORP. The former included one-on-one exit interviews with the scholars and mentors, as well as open-ended questions on surveys administered to scholars and representatives from our community organization partners about their overall experience and satisfaction with the program. The quantitative evaluation, which is the focus of this paper, was based, in large part, on the results of the Clinical Research Appraisal Inventory (CRAI), augmented by a comprehensive module on PCOR and CER skills and competencies developed by PCORP faculty. The CRAI is an 88-item self-report questionnaire specifically designed to assess research self-efficacy and the unique clinical research skills required of physician and clinician scientists across 10 domains [20]. The inventory was tested in racially diverse academic clinical researchers across five academic ranks, with a median Cronbach’s coefficient alpha of 0.96 and ANOVA eta squared effect size estimates ranging from 0.05 to 0.14.

Based on the literature, we added 14 items constructed in the same format as other CRAI items to measure specific PCOR and CER competencies not covered by the CRAI [21,22]. We excluded 50 CRAI items that were not pertinent to the content of PCORP, bringing the total number of items in the tailored instrument to 52. At the beginning, midpoint, and conclusion of a PCORP cycle, scholars rated their level of confidence in performing the general and PCOR and CER-specific tasks on an 11-point scale where 0 represents no confidence and 10 indicates complete confidence in their ability to successfully perform the task. Lastly, a series of questions asked scholars about their views on the value of and satisfaction with PCORP. We also tracked administrative data such as program completion and submitted and published manuscripts, abstracts, posters, and presentations.

### Analysis

Responses to the tailored CRAI from three cohorts were combined into one data set for a total sample of 21 matched responses (cohort 1 = 9, cohort 2 = 7, and cohort 3 = 5). Only matched responses from all three questionnaire administrations, namely, beginning (T1), midpoint (T2), and conclusion (T3), were included in the analysis. SPSS was used to conduct a Within Sample T-test to compare each of the 52 item scores and 10 subscale scores of the CRAI at all 3 administrations, including between T1 and T2, T1 and T3, and T2 and T3. A two-tailed p-value of p ≤ 0.01 was used to test for significant mean differences.

## Results

### Scholars and Projects

PCORP originally sought to train 24 scholars; however, we were able to expand to 31 scholars in the first 3 cohorts. Three scholars left the program due to position changes, making their projects no longer viable. PCORP attracted physicians, nurses, pharmacists, PhD researchers from the social sciences and psychology, and master’s level health professionals in a variety of positions (e.g., evaluation, quality improvement). In many cases, their projects were integrated into their job responsibilities, which further enhanced their experiential training. Table 2 shows the breadth and diversity of selected PCORP-supported projects, as well as their relevance to health care delivery, one of the major objectives of PCORP. Of the 22 scholars who completed the post-evaluation, 23% completed their projects before the training ended, 64% were still working it, and 9% did not plan on completing their projects. Some barriers identified to completing the project included institutional issues, electronic heath record data extraction issues, patient attrition, Institutional Review Board issues, shifts in organizational priorities, change of roles, and lack of a home institution mentor due to turnover or role transitions. The projects also benefited the home institution by engaging clinicians, generating data-driven reports and manuscripts for the institution, gaining research support and knowledge, expanding data about gaps for patient populations such as immigrants and refugees, and strengthening networks between research and practice departments, to name a few. Within 4 years of the program’s conclusion, scholars had published 22 articles and abstracts and produced 42 posters and presentations either directly linked to their PCORP training or applied to new areas beyond their PCORP-supported projects.

### Skills and Competencies

Overall, 21 out of 28 scholars (75%) completed the CRAI. Table 3 displays the means and significance for 10 CRAI subscales combined across the 3 cohorts and the 14 additional competencies and Table 4 displays the 95% confidence intervals for the 14 additional competencies. Except for the Protect Subjects and Responsible Conduct of Research subscale, all mean domain scores improved more from the beginning to the program midpoint (T1 to T2) than from the midpoint to the conclusion of the program (T2 to T3). Across the entire duration of PCORP, mean scores on all 52 items (100%) of the tailored CRAI increased significantly (*p* ≤ 0.01).

**Table 3.** *Mean scores on CRAI items and PCOR and CER skills and competencies measures* (n = 21)

CRAI subscales^±^				
	Beginning T1**	Midpoint T2**	Conclusion T3**	T1 to T3 Change
Conceptualize a study (6 items)	7.03	8.04	8.46	1.43*
Design a study (7 items)	6.03	7.54	8.15	2.12*
Collaborate with others (2 items)	6.50	8.12	8.55	2.05*
Fund a study (3 items)	5.35	6.73	7.27	1.92*
Plan and manage a study (1 item)	6.00	7.24	7.71	1.71*
Protect subjects and responsible conduct of research (3 items)	6.51	8.00	7.92	1.41*
Collect, record, analyze data (3 items)	5.40	6.73	7.60	2.21*
Interpret data (4 items)	6.45	7.48	8.13	1.68*
Report a study (6 items)	6.52	7.60	8.08	1.56*
Present a study (3 items)	6.92	8.17	8.37	1.44*
Total score for 38 general items	6.51	7.80	8.30	1.79*
Additional 14 PCOR and CER course competencies	T1	T2	T3	T1 to T3 Change
Describe health services research, evidence-based medicine, historical and regulatory evolution	4.81	7.57	8.05	3.24*
Articulate methods, findings, implications	4.71	7.62	8.10	3.38*
Critically appraise studies	4.48	7.33	7.86	3.38*
Identify data sources and their strengths and weaknesses	4.62	7.48	8.10	3.48*
Describe methods used	4.48	7.38	8.05	3.57*
Apply study results to clinical domains	4.71	7.29	8.05	3.33*
Describe quality metrics and use in health care and payment models	4.81	7.38	8.43	3.62*
Identify diverse stakeholders and describe their role in implementation and dissemination	5.57	7.95	7.81	2.24*
Describe best practices for engaging patients and stakeholders	4.71	7.57	8.14	3.43*
Identify funding sources for research and aspects of grantsmanship that differ from traditional research	3.95	7.00	7.33	3.38*
Discern between patient-, clinician-, and observer-reported outcomes	5.62	7.76	8.57	2.95*
Discuss role of patients/stakeholders in determining outcome relevance	5.29	7.86	8.14	2.86*
Describe methods used and importance of measure validity in outcome and quality measurement	4.81	7.48	7.81	3.00*
Define and provide examples of effective delivery of dissemination and implementation interventions	4.86	7.33	8.00	3.14*
Total Score for 14 PCOR and CER Items	4.82	7.50	8.03	3.21*
Total Score for All 52 Items	5.93	7.58	8.07	2.14*

CRAI, Clinical Research Appraisal Inventory; PCOR, patient-centered outcomes research; CER, comparative effectiveness research; PCORP, Patient-Centered Outcomes Research Partnership.

* Change significant at the *p* ≤ 0.01 level;

** T1 – at the beginning of PCORP program; T2 – end of year 1, approximately 1 year after T1; T3 – end of program, 1 year after T2

± Self-reported confidence scale of 0–10, where 0=none and 10=total).

**Table 4.** *Confidence intervals for PCOR and CER skills and competencies measures* (n = 21)

Additional 14 PCOR and CER course competencies	T1 vs. T3	
	95% confidence interval of the difference	
	Lower	Upper
Describe health services research, evidence-based medicine, historical and regulatory evolution	−3.54	−2.56
Articulate methods, findings, implications	−3.74	−2.66
Critically appraise studies	−3.69	−2.71
Identify data sources and their strengths and weaknesses	−3.93	−2.67
Describe methods used	−4.00	−2.80
Apply study results to clinical domains	−3.82	−2.48
Describe quality metrics and use in health care and payment models	−4.37	−2.63
Identify diverse stakeholders and describe their role in implementation and dissemination	−3.06	−1.89
Describe best practices for engaging patients and stakeholders	−3.97	−2.43
Identify funding sources for research and aspects of grantsmanship that differ from traditional research	−4.07	−2.24
Discern between patient-, clinician-, and observer-reported outcomes	−3.78	−1.60
Discuss role of patients/stakeholders in determining outcome relevance	−3.54	−1.66
Describe methods used and importance of measure validity in outcome and quality measurement	−3.71	−1.89
Define and provide examples of effective delivery of dissemination and implementation interventions	−3.85	−1.95

PCOR, patient-centered outcomes research; CER, comparative effectiveness research.

Overall, 32% of scholars reported that PCORP improved their skills significantly and 59% reported moderate improvement. PCORP’s overall usefulness to the scholars personally was rated as very good by 36%, as good by 46%, and as fair by 18%. Satisfaction with the overall experience in PCORP was rated as very satisfied by 32%, satisfied by 59%, and dissatisfied by 9%. Scholars strongly agreed/agreed that the three program components that contributed most to their research skill development were their individual project, the Summer Institute, and peer networking (90.48%, 80.95%, 80.95%, respectively). The academic mentor was also highly rated at 76.19% as were the online learning activities (76.2%, and 77.2%, respectively). Ratings for individual faculty and presenters varied considerably (data not shown). The organizational mentors, though generally rated well, were not as highly rated as other PCORP components, likely because of turn over and limited capacity to provide scholar’s support.

## Discussion

PCORP addressed the aims of the R25 funding opportunity announcement to build national capacity in the scientific workforce, expand capacity for conducting PCOR and CER studies into new fields, address health disparities, and form meaningful partnerships with organizations committed to PCOR and CER to inform health care decisions[6]. We collaborated with five partner organizations in five states, spanning thousands of miles, with striking differences in organizational structure, patients served, function, stakeholders, and philosophy. Our strategies to address training needs of future PCOR and CER practitioners involved primarily online approaches for basic training, augmented by the in-person Summer Institute and on-site experiential training through the pilot projects. By centralizing the learning environment and engaging an interdisciplinary faculty, we trained a diverse cohort of trainees with different interests and training needs while offering our investigators from partner community health systems much needed access to statistical and methodological training. These approaches are concordant with the Citizen Science initiatives that also promote public involvement in the development and direction of the scientific process [23].

Although we used a tailored version of the CRAI that cannot be directly compared to other studies, it demonstrated that PCORP was effective in training and educating scholars about PCOR and CER across many domains. CRAI scores increased most between the first and second year likely due to the intensive training in the Summer Institute, which focused on areas that the CRAI measures. These included self-reported improvements related to PCOR and CER methods, such as articulating methods, critically appraising studies, identifying data sources, describing methods, and describing quality metrics. Our program also improved other essential domains such as designing and implementing a study, collaborating with others, and collecting, recording, and analyzing data, thereby providing a strong foundation for scholars’ future PCOR and CER and evaluation endeavors.

Our focus on Native groups posed unique challenges, not only due to distances, but also because this R25 program neither supported scholars’ travel to training sites nor compensated them for time spent on PCORP pilot projects. PCORP required the home institution to support 10% of the scholars’ effort to conduct their pilot projects, rather than simply provide release-time to scholars for training. For organizations primarily focused on clinical care, especially those serving Native peoples, training programs that do not offer such support can be exigent. In addition, at Southcentral Foundation, challenges resulted from the central and vital role of customer-owners, as their potential contributions to research and the impact of the research on the organization are assessed for each project, thereby extending the time needed to complete scholars’ projects.

We encountered several organizational-level challenges. Rapidly responding to changes in organizational priorities were difficult, such as losing an orthopedic project when collaborators at a scholar’s home institution were no longer interested in her topic (that they had both suggested and endorsed at the time of application). More widespread organizational problems included shifting priorities in busy health care systems and personnel turnover including changing roles for PCORP scholars. The 2-year period for project deliverables was too long for organizations to wait for answers that they deemed pressing and important to the organization. Barriers to project completion and challenges in stakeholder engagement in PCOR research have been noted by others[24]. Furthermore, our organizational partners were often more interested in evaluating health programs or quality improvement efforts than in conducting purely research projects. Lastly, limited resources and lack of trained investigators can hinder the transition to becoming a learning health system for community health care organizations [11]. In this regard, collaboration with the Clinical and Translational Science Awards of the National Center for Advancing Translational Science training programs, which are committed to collaborative education and integrating patient perspectives into translational research, could be fruitful [25].

More generally, we recognized that addressing complex changes in health care delivery requires building sustainable infrastructures for community–academic partnerships and including patients and stakeholders in the organization and community [26]. Consistent with the emerging focus on training in learning health systems [17,19], we enriched our Summer Institute curriculum with sessions on program evaluation and quality improvement, which augmented our scholars’ skills and the value of the program to partner organizations. PCOR and CER training also should include sessions on social determinants of health and health care and vulnerable populations, which could engage diverse communities and researchers [27]. Challenges included scholar turnover and retention and scholars’ prioritization of institutional responsibilities often over PCORP deadlines and milestones; the time allotted for the PCORP program was also likely inadequate. Anecdotally, strengthening PCORP and organizational mentor relationships and improving mentor–mentee communication facilitated project success by addressing barriers early. For instance, the work begun by PCORP scholars at the Southcentral Foundation has continued for 8 years with new capacity-building efforts being undertaken under their Native American Research Centers for Health (NARCH) award [NIH grant # 5S06GM142122-02, Building Capacity for Dissemination and Implementation Research in a Tribal Healthcare System].

Several key lessons for future PCOR and CER training efforts emerged from our experience and appear consistent with the move to a learning health systems paradigm [16,28]. First, aligning the experiential project with the scholar’s and organizational goals and priorities is crucial. Buy-in at the top level of an organization is essential; projects that motivate scholars but are not immediately relevant to their organizations will flounder. PCOR and CER investigators should identify ways to better adapt research methods to health system needs, such as by creating adaptive and nontraditional experiential learning experiences. Second, the time frames for mandated projects and for achieving training objectives are not always synchronous, and organizational priorities can change, resulting in lack of completion of projects and suboptimal utilization of the experiential training portion of the program. It is, therefore, important that the scholar and academic and organizational mentors establish a structured training and project execution plan after the intensive Summer Institute to maintain the learning trajectory. Narrowing the focus of a research question and augmenting work already in progress at organizations were other successful strategies to improve project yield. Third, while online learning can be useful, the skill application through the individual project and in-person learning at the Summer Institute and peer networking were identified by scholars as contributing to their research skills development in PCOR. We recommend that future workforce development efforts explore hybrid models, such as those developed during the pandemic that integrate in-person with online learning, capitalize on advances in virtual meeting technologies, and provide investigators with the requisite skills to identify and address knowledge gaps. A fourth challenge is having adequate programming and statistical support for scholars. Most community-based clinicians and health care professionals will not succeed in rigorous research or program evaluation efforts without data-focused resources – even after obtaining sufficient skills and knowledge to lead PCOR and CER projects.

In summary, given the evidence gaps, heterogeneous health and clinical systems, and myriad decision-making challenges in the complex US health care system, strategies to improve clinical and health care system decision-making based on high-quality evidence are needed. Creating adaptive, hands-on, and nontraditional experiential learning experiences is an important pathway to support resource-constrained health systems managing challenging and competitive environments. Investigators and health systems that conduct PCOR and CER need to know how to identify, recruit, and collaborate with stakeholders to identify priorities; conceive critical questions and develop hypotheses; design, conduct, and disseminate observational and experimental research; and transform knowledge into practical applications in dynamic clinical settings. Developing these skills requires multidisciplinary training, practical experience, and strong mentorship. However, the literature on how to achieve these desirable training outcomes is evolving and the number of sufficiently qualified PCOR and CER investigators continues to be insufficient[29]. The absence of intensive, high-quality training has been recognized as a major barrier to achieving a critical mass of investigators trained in rigorous PCOR and CER methodologies[22,30]. The PCORP program offers one example of how a health services research community can work with community health systems to expand capacity and provide training for future leaders in PCOR and CER.
